# The effect of experimental pain on short-interval intracortical inhibition with multi-locus transcranial magnetic stimulation

**DOI:** 10.1007/s00221-019-05502-5

**Published:** 2019-03-27

**Authors:** Karita S.-T. Salo, Selja M. I. Vaalto, Lari M. Koponen, Jaakko O. Nieminen, Risto J. Ilmoniemi

**Affiliations:** 1 Department of Neuroscience and Biomedical Engineering, Aalto University School of Science, AALTO, P.O. Box 12200, 00076 Espoo, Finland; 2 BioMag Laboratory, HUS Medical Imaging Center, University of Helsinki and Helsinki University Hospital, Helsinki, Finland; 3 Department of Clinical Neurophysiology, HUS Medical Imaging Center, University of Helsinki and Helsinki University Hospital, Helsinki, Finland; 4 Department of Psychiatry and Behavioral Sciences, School of Medicine, Duke University, Durham, NC USA

**Keywords:** Transcranial magnetic stimulation, Motor cortex, Acute pain, Motor-evoked potential, Neuropathic pain

## Abstract

Chronic neuropathic pain is known to alter the primary motor cortex (M1) function. Less is known about the normal, physiological effects of experimental neurogenic pain on M1. The objective of this study is to determine how short-interval intracortical inhibition (SICI) is altered in the M1 representation area of a muscle exposed to experimental pain compared to SICI of another muscle not exposed to pain. The cortical representation areas of the right abductor pollicis brevis (APB) and biceps brachii (BB) muscles of 11 subjects were stimulated with a multi-locus transcranial magnetic stimulation device while the resulting motor-evoked potentials (MEPs) were recorded with electromyography. Single- and paired-pulse TMS was administered in seven conditions, including one with the right hand placed in cold water. The stimulation intensity for the conditioning pulses in the paired-pulse examination was 80% of the resting motor threshold (RMT) of the stimulated site and 120% of RMT for both the test and single pulses. The paired-pulse MEP amplitudes were normalized with the mean amplitude of the single-pulse MEPs of the same condition and muscle. SICI was compared between conditions. After the cold pain, the normalized paired-pulse MEP amplitudes decreased in APB, but not in BB, indicating that SICI was potentially increased only in the cortical area of the muscle subjected to pain. These data suggest that SICI is increased in the M1 representation area of a hand muscle shortly after exposure to pain has ended, which implies that short-lasting pain can alter the inhibitory balance in M1.

## Introduction

Chronic neuropathic pain is known to alter the functioning of the motor and sensory systems, for example, by inducing reorganization in the primary somatosensory cortex (S1) (Pleger et al. 2004; Vartiainen et al. 2009). The extent of cortical reorganization is related to the intensity of pain (Flor 2003). Patients with complex regional pain syndrome (CRPS) have been reported to have a decreased distance between the different finger representation areas of the painful hand in S1 (Vartiainen et al. 2009). In addition, expansion (Pleger et al. 2004) and disinhibition (Lenz et al. 2011; Schwenkreis et al. 2010) of the representation areas of the painful hand in S1 have also been shown. Disinhibition (Chang et al. 2018; Lefaucheur et al. 2006) and expansion (Flor 2003) of the representation area of the painful limb have been found in the primary motor cortex (M1) of patients with CRPS and other chronic neuropathic pain conditions. The underlining mechanisms of this maladaptive neuroplasticity are not yet well known (Flor 2003). However, it has been shown that multiple factors, such as gender (Bartley and Fillingim 2013) and genetic factors, affect the generation of chronic pain; for example, a specific dopamine receptor genotype predisposes people to chronic neuropathic pain (Jääskeläinen et al. 2014).

Surround inhibition regulates the somatotopic organization and production of fine motor movements in M1 (Beck et al. 2008) and sensory discrimination in S1 (Mountcastle and Powell 1959). One indicator of surround inhibition is gamma-aminobutyric acid A-mediated short-interval intracortical inhibition (SICI) (Hanajima et al. 1998; Kujirai et al. 1993; Ziemann et al. 1996). In chronic pain, tactile acuity has been reported to decrease in some patients, such as with CRPS, but not change in others like those with burning mouth syndrome (Catley et al. 2014). In addition, Strauss et al. (2015) showed that anesthetic cream improved muscle function in CRPS patients implying that disinhibition was normalized in the sensorimotor cortex.

The inter- and intracortical facilitatory and inhibitory mechanisms, such as SICI, in M1 can be studied with the combination of paired-pulse transcranial magnetic stimulation (TMS) (Barker et al. 1985; Di Lazzaro et al. 1998, 2013) and electromyography (EMG) (Ferbert et al. 1992; Hallett 2000; Kujirai et al. 1993). The paired-pulse paradigm for SICI consists of a subthreshold conditioning stimulus and a suprathreshold test stimulus with a 1–6-ms interstimulus interval (ISI) (Ilić et al. 2002; Kujirai et al. 1993; Ziemann et al. 1996). In this paradigm, the amplitude of the resulting paired-pulse motor-evoked potential (pp-MEP) is decreased compared to that of a single-pulse MEP (sp-MEP) after a suprathreshold test stimulus alone (Kujirai et al. 1993).

In a review of studies with neuropathic and non-neuropathic pain patients, Chang et al. (2018) reported that chronic pain does not alter SICI. However, in a subgroup analysis, a relation between a moderate decrease in SICI and CRPS was found (Chang et al. 2018; Lefaucheur et al. 2006; Schwenkreis et al. 2010). In chronic non-neuropathic pain, long-interval intracortical inhibition was increased (Chang et al. 2018), whereas SICI was decreased in patients with lateral epicondylalgia (Burns et al. 2016a). The controversial results in SICI between studies with chronic pain patients could be explained by the fact that some non-neuropathic pain conditions also include a neurogenic pain component similar to those in neuropathic pain complicating the interpretation of the results (Groppa 2016; Spahr et al. 2017). The effect of experimental pain on SICI in healthy volunteers has been studied before with divisive results; tonic pain increased SICI afterward (Schabrun and Hodges 2012), but capsaicin-induced pain decreased SICI during pain (Fierro et al. 2010).

We investigated how acute experimental pain, mimicking neuropathic pain (short-lasting cold pain), affects SICI and how local the effect is. We aimed to determine how SICI is altered in the M1 representation area of the muscle exposed to experimental pain compared to SICI of a remote upper limb muscle not exposed to the experimental pain in healthy individuals. If SICI was altered only at the representation area of the exposed muscle, it might indicate a normal, very local, physiological response to acute pain in M1, indicating that adaptive functional changes are related to acute pain and that these are distinct from maladaptive changes in chronic pain. Cold water was chosen to induce acute pain since it is easy to execute and suitable for TMS studies. Schabrun and Hodges (2012) have studied two nearby hand muscles in an experimental-pain study by stimulating only the optimal representation area of the other muscle since the M1 representation areas of these muscles are very near and often overlap. We stimulated the two sites in M1 with a multi-locus TMS (mTMS) device that allows shifting the stimulation target electronically without physically moving the coil (Koponen et al. 2018a), allowing us conveniently to give TMS pulses in a random order to two nearby sites in M1. This randomization minimizes changes unrelated to the experimental manipulation when comparing two muscles.

## Materials and methods

### Subjects

Eleven volunteers (nine right-handed; six males; mean age 31 years, range 26–39) participated in the experiment after giving their written informed consent. Compliant with the Declaration of Helsinki, the study was approved by the Coordinating Ethics Committee of the Hospital District of Helsinki and Uusimaa.

### TMS and EMG

First, we obtained a T1-weighted magnetic resonance image of the head of each subject to allow neuronavigation (Nexstim eXimia, Nexstim Plc, Helsinki, Finland). Then the subjects were stimulated with an mTMS device (Koponen et al. 2018a) while the measure of MEPs from the right abductor pollicis brevis (APB) and biceps brachii (BB) was recorded with a Nexstim eXimia EMG device using surface electrodes. The active electrodes were placed on top of the muscles; for APB, the reference electrode was placed on the first metacarpophalangeal joint and for BB on the tendon inside the elbow. The ground electrode was placed on the forearm approximately halfway between the right APB and BB. The APB electrodes were covered with waterproof tape.

Stimulation targets for the right APB and BB in the left M1 were determined by finding the cortical locations that produced the highest MEP amplitudes in the respective muscles for single-pulse monophasic TMS was administered with a figure-of-eight coil; the first phase of the induced current was in the posterior–anterior (PA) direction. The stimulation site for BB was found by locating a stimulation target that produced larger amplitudes for BB MEPs than for APB MEPs if a stimulation target with only BB MEPs was not found. The Euclidean distance between the two cortical targets was calculated from the information provided by the neuronavigation system. The mTMS transducer was subsequently kept fixed above the APB target, and the targeting for APB and BB was done electronically. The resting motor threshold (RMT) was defined separately for the two muscles as the lowest stimulation intensity producing an MEP with an amplitude of at least 50 µV in at least 10 out of 20 consecutive trials (Rothwell et al. 1999) with an ISI of 4–6 s. The RMT of APB was always found before that of BB. The RMT was determined for a trapezoidal pulse with a 60-µs-long initial phase of the induced electric field in the PA direction, followed by a 30-µs-long phase of the near-zero electric field and a 44.8-µs-long phase of the electric field oriented from anterior to posterior, in a manner similar to that in our previous experiment (Koponen et al. 2018b).

**Fig. 1:**
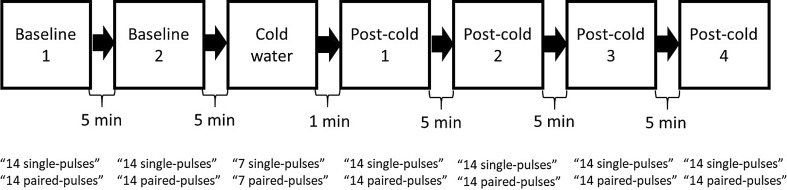
A diagram showing the chronological order of the sequences

Single and paired pulses were given in seven stimulation sequences (Fig. 1). Each sequence contained 14 single pulses and 14 paired pulses (conditioning and test pulses with a 2.5-ms ISI) to both targets with a 3-s intertrain interval (ITI), except for the third sequence, which included exposure to cold pain (with at most seven single and seven paired pulses to both targets to limit the maximum duration of cold exposure to 90 s). The first two conditions were baselines, and the last four are here referred to as post-cold conditions. The stimulation sequences were separated by 5-min intervals (except for a 1-min interval between the third and fourth conditions, that is, the cold pain and the first post-cold conditions). Two stimulus sequences were given in the baseline condition to learn about MEP variability over time.

The stimulation intensity was 80% of RMT of the stimulated site for the conditioning pulses and 120% of RMT for both the test and single pulses. The 80% and 120% intensities, for all conditions and for both single and paired pulses, were created by adjusting the duration of the trapezoidal pulses so that the depolarization of a cell membrane with a 200-µs strength–duration time constant (Peterchev et al. 2013) would be 80% or 120% of the pulses used for determining the RMT. In practice, this meant that the 80% of the RMT pulses had an initial PA-oriented electric field with a duration of 43.2 µs, and the 120% of the RMT pulses had an initial PA-oriented electric field with a duration of 81.2 µs (the duration of the near-zero electric field was at 30 µs; those of the final phases were 33.2 and 58.7 µs, respectively). The order of the pulses (single and paired) and the order of the stimulated targets were randomized. The time between the baseline sequences and the cold condition was 19 min longer for six subjects because two additional baseline conditions and a warm-water condition with breaks were applied to them. With the warm-water condition, we aimed to verify that the sensory stimulus of water by itself did not alter SICI; however, these data were not included for further analyses because the data of two subjects were corrupted due to electrode contact with water. It is doubtful that the warm water would have affected the results since there was 19 min between the warm-water and the cold conditions. For these two subjects, the faulty waterproofing of the electrodes was mended before their respective cold-water sequences.

### Cold pain

In the cold-pain condition (the third condition), subjects placed their right hand into cold water (2–4 °C). Only APB was exposed to the cold, while BB provided a reference. Subjects were instructed to take their hand off the water if they felt intolerable pain or, at the latest, after 90 s of exposure. Every 10 s, with their left hand, they pointed at a number to indicate the intensity of the pain on a numerical rating scale (NRS) ranging from zero to 10, zero meaning “no pain” and 10 being “the most intensive imaginable pain”. The subjects were also asked to quantify the level of pain before and after the other conditions.

### Analysis

The EMG data were visually checked to ensure that the MEP amplitudes were correctly determined by the analysis software. Some trials were removed due to involuntary muscle preactivation. If no MEP was visible, the corresponding amplitude was set to zero. The responses from each condition were divided into four categories: single or paired pulses to APB or BB. The paired-pulse data of each subject were normalized by dividing the MEP amplitudes by the mean of the single-pulse amplitudes from the same muscle and condition. For each condition, the normalized responses were merged across subjects.

First, we compared the two baseline conditions. According to a two-tailed permutation test, there was no significant difference between the normalized pp-MEP amplitudes of APB in the first and the second baseline conditions (difference 0.05 ± 0.57 (mean ± standard deviation); *p* = 0.38, uncorrected, two tailed; the test was similar to the other tests applied in this study, see below). Thus, the baselines were combined and the combination, referred to as “the baseline” from now on, was applied to further analyses. To quantify the difference in the normalized pp-MEP amplitudes between the baseline and the cold- and post-cold conditions, we computed the difference in the corresponding mean amplitudes. Two-tailed permutation tests were performed to assess the statistical significance of these differences. Five permutation tests were done separately for the normalized pp-MEP amplitudes of APB and BB. The first test was run between the baseline and the cold condition and the second between the baseline and the first post-cold condition. The third was executed in the same way between the baseline and the combination of the first and second post-cold conditions. The fourth test was run between the baseline and the combination of the first three post-cold conditions and the fifth test between the baseline and the combination of all the post-cold conditions.

A nonparametric statistical test was chosen because the responses could not be assumed to be normally distributed. First, we calculated the difference between the means of the normalized MEP amplitudes of the selected two conditions. Next, we generated 100,000 datasets in each of which the data points from the two conditions were randomly assigned to produce two response sets of the same size as the originals. For each such dataset, we computed the difference of the mean MEP amplitude of the two response sets. To obtain a two-tailed *p* value for the null hypothesis of no difference between the two conditions, we counted the number of randomized datasets for which the absolute value of the difference exceeded the absolute value of the difference in the original data and divided this value by 100,000. The permutation tests were not adjusted for multiple comparisons because the datasets for the tests overlapped with each other; thus, being dependent on each other in different ways. The Spearman correlation coefficients between the maximum NRS value of each subject and their mean normalized pp-MEP amplitude in the cold condition were also computed for APB and BB. Power calculations for normalized data (Kadam and Bhalerao 2010) were derived according to an earlier study with a similar experiment (Schabrun and Hodges 2012).

## Results

The distance of the identified APB and BB targets in the cortex varied from 5 to 14 mm (mean 10.5 mm) across subjects. For each subject, the BB target in the precentral gyrus was more medial than the APB target. The ratio of the BB and APB RMT was 1.2 ± 0.1 (mean ± standard deviation across subjects). Less than 1% (31/3996) of the trials were removed due to preactivation. One of the subjects kept her hand in the water for only 70 s as the pain had reached the NRS value of 10; the remaining subjects completed the full 90 s. The temperature of the water rose approximately 1.5° during the cold-pain condition.

The maximum NRS values can be seen in Table 1. The Spearman correlation coefficient between the maximum NRS value and the mean normalized pp-MEP amplitude in the cold condition was − 0.005 for APB and 0.32 for BB; thus, there was no clear correlation between the NRS values and SICI. The reported NRS values were 0 before and after all the other conditions for all but two subjects who reported an NRS value of 1 because of pain caused by the navigation goggles or neck pain before the cold condition but not after.

**Table 1:** The number of subjects with each of the maximum NRS values

Maximum NRS value	Number of subjects
3	1
4	1
5	3
6	2
9	2
10	2

**Fig. 2:**
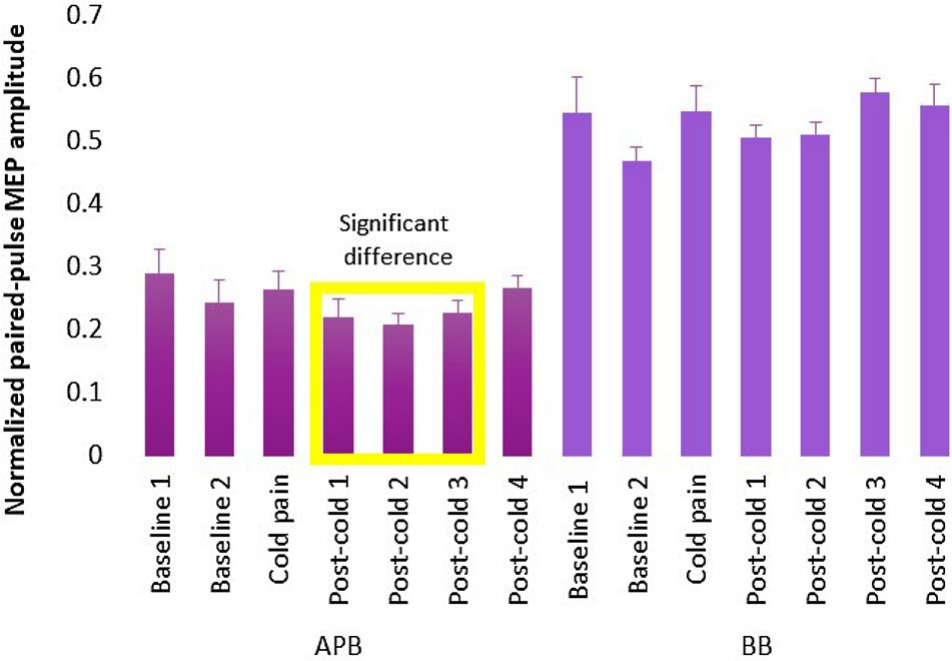
Normalized pp-MEP amplitudes for the baseline and the other five conditions measured from APB and BB averaged across subjects. The error bars indicate the standard error of mean across subjects. The yellow rectangle highlights the conditions with a significant difference compared to the baseline for APB

The normalized post-cold pp-MEP amplitudes of APB first decreased before returning to the baseline level, suggesting an increase in SICI right after the end of the exposure to cold pain (Fig. 2). The largest decrease (29%) in normalized pp-MEP amplitudes compared to the baseline was in the second post-cold condition 6 min after the end of the cold exposure. The corresponding *p* values and mean effect sizes are reported in Table 2. No significant difference between the baseline and the post-cold conditions was observed for BB (Fig. 2; Table 2). The power calculations (*α* = 0.05, *β* = 0.80, effect size = 0.5, standard deviation = 32) resulted in 32 samples.

**Table 2:** The uncorrected *p* values and the mean effect size in percentages between the compared conditions for the two-tailed permutation tests

The baseline compared to	*p* value for APB	Mean effect for APB (%)	*p* value for BB	Mean effect for BB (%)
Cold condition	0.394	− 17.4	0.685	− 5.30
First post-cold condition	0.252	18.0	0.953	0.50
Combination of first and second post-cold conditions	0.036	23.7	0.880	− 1.00
Combination of first, second, and third post-cold conditions	0.033	20.7	0.362	− 5.30
Combination of all four post-cold conditions	0.078	15.6	0.244	− 6.40

## Discussion

We found that normalized pp-MEP amplitudes of the distal hand muscle (APB) were reduced shortly after the exposure to painful cold water. This indicated that SICI was increased in the corresponding M1 representation area. No such change was seen in the M1 representation area of the proximal hand muscle (BB) that was not exposed to cold. Our results are in line with a previous study by Schabrun and Hodges (2012) who found that SICI increased after but not during tonic pain. However, there are also studies with opposite results. Mavromatis et al. (2016) reported that pp-MEP amplitudes increased and SICI decreased after experimental pain (with cuff inflation). In addition, Schabrun et al. (2016) demonstrated that acute muscle pain following developing muscle soreness caused an increase in the representation area of the muscle exposed to pain, which was related to decreased SICI and increased intracortical facilitation in M1. Other physiological changes related to acute pain have also been reported, such as a decrease in corticomotor output and cortical excitability (Burns et al. 2016b). Based on previous studies and our study, SICI alterations might be reversed during pain and after pain; increased inhibition after pain might be a homeostatic response to the decreased SICI during pain.

A moderate decrease in SICI has been shown to be related to CRPS, which may involve the dysfunction of the limb (Chang et al. 2018; Lefaucheur et al. 2006; Schwenkreis et al. 2010). However, Chang et al. (2018) concluded in their review that there is no correlation between altered SICI and chronic pain when studies with non-neuropathic and neuropathic pain patients were combined. The differing results in SICI between studies with chronic pain patients might be explained by the fact that typical non-neuropathic pain conditions might also have some neurogenic component producing results with altered and unaltered SICI depending on the type of pain. An increase in SICI after acute pain might be necessary to avoid cortical reorganization and, if long lasting, the dysfunction of the limb as is in CRPS. It is also possible that an increase in SICI after cold pain is a homeostatic feedback mechanism to the decreased SICI during the cold pain even if we could not show this disinhibition shown in another study (Schabrun et al. 2016).

The experimental pain was generated with cold water to gain a long-lasting and continuous pain condition for SICI measurements. To confirm that the altered SICI was related to the pain, it would have been beneficial to study the function of the nociceptive pathway before, during, and after the cold-water exposure, for example, with laser-evoked potentials (LEPs). Previous studies have shown that LEPs are inhibited by simultaneous non-noxious somatosensory stimulation (Testani et al. 2015). Also, it has been shown that preceding noxious stimuli modulate the following TMS-generated MEP amplitudes (Valeriani et al. 1999), which could have been one way to study alterations in the function of the nociceptive pathway beside LEP recordings. In our study, the noxious stimuli transmitted via the spinothalamic pathway were combined and competed with the sensation of cold and water-generated somatosensory stimuli; thus, the altered SICI might have been caused by the combination of different stimuli and not just the noxious stimuli. Using another pain-producing method and observing changes in SICI could have confirmed that noxious stimuli themselves had an effect on SICI.

One crucial limitation in this study was the number of subjects. According to the power calculations, 32 samples would have been appropriate for this kind of study. However, the power calculations were derived for normalized data and might not be easily applied to permutation tests. The sample size was larger than the number of subjects since each normalized pp-MEP was used separately and not just the mean of MEPs from each subject. Nevertheless, we observed a clear and consistent trend of increased SICI lasting for several consecutive post-cold sequences with the small subject number in this pilot study.

There have been investigations of the effects of acute experimental pain on SICI and other M1 functions in the representation areas of the painful and remote muscles, such as a study of Schabrun and Hodges (2012), which showed that SICI increased after pain in the painful muscle but not in the nearby muscle. Farina et al. (2001) showed a trend of decreased sp-MEP amplitudes in the surrounding hand and forearm muscles of the painful muscle and Svensson et al. (2003) that the sp-MEP amplitudes were not suppressed in a remote muscle not exposed to pain. However, it is known that altered SICI is not straightforwardly related to changes in the amplitudes of sp-MEPs (Quinn et al. 2018). We introduced a new experimental setting to study SICI from two nearby M1 representation areas. With the mTMS device, we were able to interleave the stimulation of two cortical locations in a randomized order in the time window given by the ethical limits to expose individuals to experimental cold pain. The randomization minimized any potential systematic build-up effects of the responses to the previous stimuli. The mTMS allowed us to study the spatial extent of changes in SICI from the distal and proximal upper limb muscle representation areas. The difference between the APB and BB responses indicates that the suggested increase in SICI was very local, as the two stimulation targets were separated only by 5–14 mm.

The experiment involved attention, as the subjects were asked to focus on the pain. Several studies have shown an increase in facilitation in the cortex (Stefan and Wycislo 2004) or increase in sp-MEP amplitudes (Mavromatis et al. 2016; Thomson et al. 2008) due to attention. In contrast, Thomson et al. (2008) demonstrated that attention did not change the normalized pp-MEPs or SICI, although it had an increasing effect on sp-MEP amplitudes. In our study, attention may have affected SICI especially in the cold-pain condition; the subjects were asked to report the level of pain every 10 s during the cold pain but were asked just before and after trials in all the other conditions.

There have also been studies highlighting the subjective nature of pain (Kucyi and Davis 2015); pain intensity has been shown to be related to the extent of cortical reorganization in S1 and M1 (Flor 2003; Karl et al. 2001; Lotze et al. 2001; Pleger et al. 2004). Also, different measures of pain, especially cold pain detection thresholds, have large inter-individual variability and the estimation of pain intensity is always subject specific and varies between subjects. We did not measure the skin temperature before and after the cold water exposure; thus, we do not know if the subjective pain intensities were related to the tissue temperature changes. However, earlier studies have shown that the pretest temperature of the skin has only a minor effect on thermal and pain detection thresholds (Hilz et al. 1999). Lotze et al. (2001) did not study SICI, but they showed an increase in the representation of the facial muscles in patients with phantom limb pain after hand amputation, indicating a decrease in surround inhibition in the representation area of the amputated upper limb and the area of reorganized facial representation was compared with level of pain. We found no clear correlation between the individual maximum NRS score and the magnitude of SICI. Regarding whether the sensation of water itself rather than cold pain alters pp-MEPs, studies have demonstrated that the sensation of water is insufficient to alter SICI (Sato et al. 2013); however, we cannot exclude the possible effect of the pure painless sensory stimulus on SICI.

## Conclusion

We found that after the cold pain, normalized pp-MEP amplitudes were smaller than before the pain in the muscle exposed to cold, suggesting that SICI is increased after cold water exposure. This finding supports the view that increased SICI is a typical physiological response after the acute phase of pain. Our study paradigm was made possible by a recently introduced mTMS device that enables one to study SICI locally from adjacent cortical locations without moving the coil.
